# Sensitivity analysis of transabdominal fetal pulse oximetry using MRI-based simulations

**DOI:** 10.1364/BOE.531149

**Published:** 2024-08-19

**Authors:** Jingyi Wu, Gopika Satish, Alexander Ruesch, Baptiste Jayet, Katarzyna Komolibus, Stefan Andersson-Engels, Martin P. Debreczeny, Jana M. Kainerstorfer

**Affiliations:** 1 Department of Biomedical Engineering, Carnegie Mellon University, 5000 Forbes Avenue, Pittsburgh, PA 15213, USA; 2 Neurolscience Institute, Carnegie Mellon University , 4400 Forbes Avenue, Pittsburgh, PA 15213, USA; 3 Biophotonics@Tyndall, Tyndall National Institute, Lee Maltings Complex, Dyke Parade, T12 R5CP Cork, Ireland; 4 School of Physicss, University College Cork, College Road, T12 K8AF Cork, Ireland; 5 Raydiant Oximetry, Inc., San Ramon, CA 94583, USA

## Abstract

Transabdominal fetal pulse oximetry offers a promising approach to improve fetal monitoring and reduce unnecessary interventions. Utilizing realistic 3D geometries derived from MRI scans of pregnant women, we conducted photon simulations to determine optimal source-detector configurations for detecting fetal heart rate and oxygenation. Our findings demonstrate the theoretical feasibility of measuring fetal signals at depths up to 30 mm using source-detector (SD) distances greater than 100 mm and wavelengths between 730 and 850 nm. Furthermore, we highlight the importance of customizing SD configurations based on fetal position and maternal anatomy. These insights pave the way for enhanced non-invasive fetal monitoring in clinical application.

## 1. Introduction

The well-being of a fetus inside the uterus relies on oxygen delivered via the umbilical cord from the mother. During delivery, maternal contractions can compress the vessels in the uterus and umbilical cord, potentially reducing oxygen flow to the fetus. Such a condition is known as fetal hypoxia, which can result in serious consequences on the developing fetal brain. Severe oxygen deprivation could lead to hypoxic-ischemic encephalopathy (HIE), which can result in long-term neurological disorders, movement disorders, and even fetal death [1]. Currently, there is no medical device capable of non-invasively measuring fetal oxygenation directly.

Fetal monitoring during delivery is primarily performed using cardiotocography (CTG), which tracks fetal heart rate (FHR) and maternal contractions. FHR is modulated by the fetal nervous system and is responsive to variations in oxygen levels [2]. However, FHR interpretation is challenging due to its sensitivity to various other physiological and measurement factors, often leading to ambiguous clinical interpretation [3]. Observation of a non-reassuring FHR is often used as the basis for performing Cesarean sections (C-sections) to prevent potential hypoxic injuries [4]. While C-sections can be lifesaving, they are major surgical procedures that increase recovery time, and pain; and carry risks such as infections and future uterine ruptures [5]. The low positive predictive rate (about 30%) and high false-positive rate (over 60%) of CTG for detecting fetal hypoxia result in many unnecessary C-sections, introducing avoidable risks [6].

To improve maternal and fetal outcomes and reduce unnecessary C-sections, transabdominal fetal pulse oximetry has been proposed. This technique utilizes near-infrared (NIR) light to non-invasively measure fetal arterial oxygen saturation (SpO_2_) directly through the maternal abdomen in reflection geometry [7,8]. However, this approach is complicated by the multi-layer tissue structure above the fetus, leading to a fetal depth from approximately 1.5 to 4.5 cm [9,10]. Questions remain about whether the light can effectively reach the fetal brain and whether pulsatile absorption changes due to fetal heartbeat can be detected and used to quantify blood oxygen saturation. While pulse oximetry in adults typically focuses on peripheral oxygen levels, the specific conditions during labor require monitoring the fetal brain to assess its susceptibility to hypoxic events. Additionally, fetuses can assume various positions during delivery, with the head typically facing down. The fetal head provides a more uniform geometry across different subjects, allowing for more consistent measurements and better comparison of results across different fetal geometries. While Monte Carlo simulations on simplified slab or spherical geometries [11–14], phantom experiments [15], and animal studies [16] have provided some insights, there is a lack of studies using realistic 3D human maternal and fetal anatomical models to analyze different wavelengths and source-detector (SD) configurations.

In this work, we utilized realistic 3D models derived from MRI scans of pregnant women. We generated 3D meshes from these models, and performed photon simulations using NIRFASTer [17,18]. Additionally, we placed an SD grid on the maternal abdomen to examine measurement sensitivities across various wavelengths and SD configurations, incorporating simulated noise to reflect more realistic conditions. These analyses aim to provide a basis for improved clinical fetal monitoring techniques.

## 2. Method

### 2.1 3D model creation from MRI scans with different fetal positions

To examine the measurement sensitivities of transabdominal fetal pulse oximetry on realistic human anatomy, we utilized 3D models derived from T2 MRI scans of pregnant women. The MRI measurements were conducted in St. Thomas’ Hospital, London, UK. Prior to the study, all maternal subjects provided their written informed consent.

In this work, we used MRI scans from two women beyond 37 weeks of gestation, representing the two common fetal head positions: Occiput Anterior (OA) and Occiput Posterior (OP). OA describes positions where the fetus faces the mother’s back with the head down, which is optimally aligned for birth. In the OP position, the fetus faces the mother’s abdomen with the head down, known for complicating labor due to adverse fetal head orientation [19]. These positions impact the sensitivity of fetal pulse oximetry due to varying depths and orientations of the fetus.

**Fig. 1.**
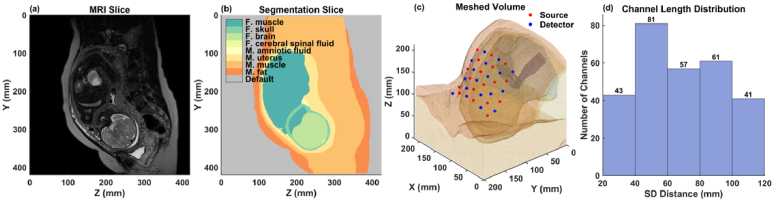
Fetus in Occiput Anterior (OA) position. (a) Sagittal MRI slice. (b) Segmentation of (a). (c) 3D mesh with SD grid on the maternal abdomen. (d) Channel length distribution of the SD grid in (c).

**Fig. 2.**
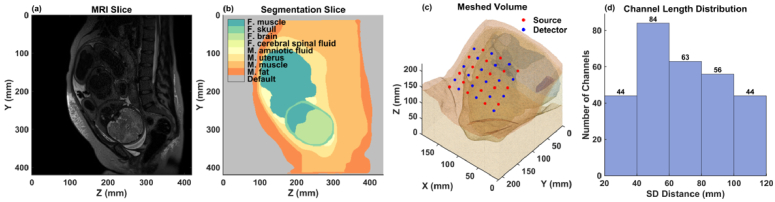
Fetus in Occiput Posterior (OP) position. (a) Sagittal MRI slice. (b) Segmentation of (a). (c) 3D mesh with SD grid on the maternal abdomen. (d) Channel length distribution of the SD grid in (c).

Using 3D Slicer [20], we segmented the maternal and fetal tissues from the MRI scans. The segmentations include maternal tissue such as fat, abdominal muscle, uterus, and amniotic fluid, and fetal tissue such as muscle, skull, brain, cerebral spinal fluid (CSF), and spinal cord. The MRI scans had a voxel resolution of 1.25 mm, and the full 3D volumes are about 430 × 430 × 300 mm. Figure 1(a-b) and Fig. 2(a-b) show example sagittal MRI slices and their corresponding segmentations for OA and OP fetal positions.

To enhance computational efficiency, we focused only on the lower torso of the maternal model that includes the fetal head. Using MATLAB (Mathworks, Inc., Natick, MA) and 3D triangulation algorithm in the Computational Geometry Algorithm Library [21], we generated a mesh containing ∼0.6 million nodes and ∼3.7 million tetrahedrons. The average voxel size was 1.82 mm^3^, which balances the need for computational efficiency with good spatial resolution. Figure 1(c) and Fig. 2(c) show the 3D meshes for the OA and OP positions, respectively, with the optical SD grid placed on the surface of the maternal abdomen.

### 2.2 Photon simulation with NIRFASTer

Photon simulations were performed using NIRFASTer, a simulation toolbox that solves light propagation in biological tissue using the finite element method. This toolbox applies the diffusion equation to model the light transportation within tissue, which is crucial for predicting the interaction between light and tissue structures [18].

For our simulation, we used the “femdata_FD” function in NIRFASTer, which calculates the fluence rate ( ϕ [s^−1^mm^−2^], normalized by source power) at each node within the mesh based on the simulated source location, providing information about the photon distribution across the meshed volume.

The subsequent sections will outline the prerequisite steps for the simulation, which include optical property setup (2.2.1) and source-detector setup (2.2.2).

#### 2.2.1 Optical property setup

To initiate the simulation, optical properties for each tissue type must be specified, including absorption coefficient ( $\mu_{a}$ ), reduced scattering coefficient ( $\mu_{s}^{\prime }$ ), and refractive index ( n ).

**Absorption coefficient**: For each tissue type, $\mu_{a}$ was calculated using [22]:

$$\begin{array}{ll} \mu_{a,\;\text{tissue}} & =[\text{HbT}]\cdot \text{St}{\text{O}}_{2}\cdot \epsilon_{\text{HbO}}+[\text{HbT}]\cdot (1-\text{St}{\text{O}}_{2})\cdot \epsilon_{\text{Hb}}+V_{\text{water}}\cdot \mu_{a,\;\text{water}}+V_{\text{fat}}\cdot \mu_{a,\;\text{fat}}\\ & +V_{\text{melanin}}\cdot \mu_{a,\;\text{melanin}}, \end{array}$$

where [HbT] is the concentration of total hemoglobin [µM] in the tissue, StO_2_ [–] is the tissue oxygen saturation, $\epsilon_{HbO}$ and $\epsilon_{Hb}$ [cm^−1^µM^−1^] are the extinction coefficients of oxy- and deoxy- hemoglobin, respectively, and *V* [–] represents the volume fraction. The absorption coefficients of water ( $\mu_{a,\;\text{water}}$ ), fat ( $\mu_{a,\;\text{fat}}$ ), and melanin ( $\mu_{a,\;\text{melanin}}$ ) are taken from [22], and parameters, including values of [HbT] and *V*, are summarized in Table 1 and Table 2 for maternal and fetal tissues, respectively.

**Table 1.** Parameters for Calculating Maternal Tissue Optical Properties

Tissue Type	[HbT] (µM)	StO_2_ (%)	$V_{\text{water}}$	$V_{\text{fat}}$	$V_{\text{melanin}}$	*a* (cm^−1^)	*b*	*n*	Ref.
Fat	12.2	75	0.23	0.72	0	9.69	0.81	1.4	[22,25]
Muscle/Uterus	26.2	75	0.76	0	0	15.42	0.89	1.4	[22,25]

**Table 2.** Parameters for Calculating Fetal Tissue Optical Properties

Tissue Type	[HbT] (µM)	SaO_2_ (%)	SvO_2_ (%)	$V_{\text{water}}$	$V_{\text{fat}}$	$V_{\text{melanin}}$	*a* (cm^−1^)	*b*	*n*	Ref.
Brain	39.7	50	40	0.76	0	0	22.52	1.56	1.3	[13,22]
Skull/Spinal cord	39.7	50	40	0.16	0	0	22.90	0.72	1.3	[22]
Muscle	33.5	50	40	0.76	0	0	15.42	0.89	1.3	[22,25]

**Simulating fetal cardiac pulse**: $\mu_{a}$ change due to fetal pulsation was simulated by increasing the [HbT] only in the arterial compartment of fetal brain and muscle. Specifically, after setting the arterial and venous oxygen saturation (SaO_2_ and SvO_2_) and assuming arterial and venous blood volumes ( $V_{a}$ and $V_{v}$ ) being 25% and 75% of the total [HbT], we can calculate arterial and venous [HbO] and [Hb] by ${\left[\text{HbO}\right]}_{a}=[\text{HbT}]\cdot V_{a}\cdot \text{Sa}{\text{O}}_{2}$ , ${\left[\text{Hb}\right]}_{a}=[\text{HbT}]\cdot V_{a}\cdot (1-\text{Sa}{\text{O}}_{2})$ , ${\left[\text{HbO}\right]}_{v}=[\text{HbT}]\cdot V_{v}\cdot \text{Sv}{\text{O}}_{2}$ , ${\left[\text{Hb}\right]}_{v}=[\text{HbT}]\cdot V_{v}\cdot (1-\text{Sv}{\text{O}}_{2})$ . Then, $\mu_{a}$ at diastolic state is defined by [23,24]:

$$\begin{array}{ll} \mu_{a,\;\text{diastole}} & =({\left[\text{HbO}\right]}_{a}+{\left[\text{HbO}\right]}_{v})\cdot \epsilon_{\text{HbO}}+({\left[\text{Hb}\right]}_{a}+{\left[\text{Hb}\right]}_{v})\cdot \epsilon_{\text{Hb}}+V_{\text{water}}\cdot \mu_{a,\;\text{water}}+V_{\text{fat}}\cdot \mu_{a,\;\text{fat}}\\ & +V_{\text{melanin}}\cdot \mu_{a,\;\text{melanin}}. \end{array}$$

For systolic state, a volume fraction increases due to fetal pulsation ( $V_{p}=$ 5%) is considered. Then, $\mu_{a}$ at diastolic state is:

$$\begin{array}{ll} \mu_{a,\;\text{systole}} & =({\left[\text{HbO}\right]}_{a}\cdot (1+V_{p})+{\left[\text{HbO}\right]}_{v})\cdot \epsilon_{\text{HbO}}+({\left[\text{Hb}\right]}_{a}\cdot (1+V_{p})+{\left[\text{Hb}\right]}_{v})\cdot \epsilon_{\text{Hb}}\\ & +V_{\text{water}}\cdot \mu_{a,\;\text{water}}+V_{\text{fat}}\cdot \mu_{a,\;\text{fat}}+V_{\text{melanin}}\cdot \mu_{a,\;\text{melanin}}. \end{array}$$

**Reduced scattering coefficient**: In cm^−1^, $\mu_{s}^{\prime }$ is modeled as a power law decay: $\mu_{s}^{\prime }=a\cdot {\left(\lambda /\lambda_{0}\right)}^{-b}$ [22], where λ is wavelength in nm, $\lambda_{0}$ = 500 nm is the reference wavelength, *a* is the value of $\mu_{s}^{\prime }$ at 500 nm, and *b* is the scattering power. Tissue specific *a* and *b* values are provided in Table 1 and Table 2 for maternal and fetal tissues, respectively.

**Fig. 3.**
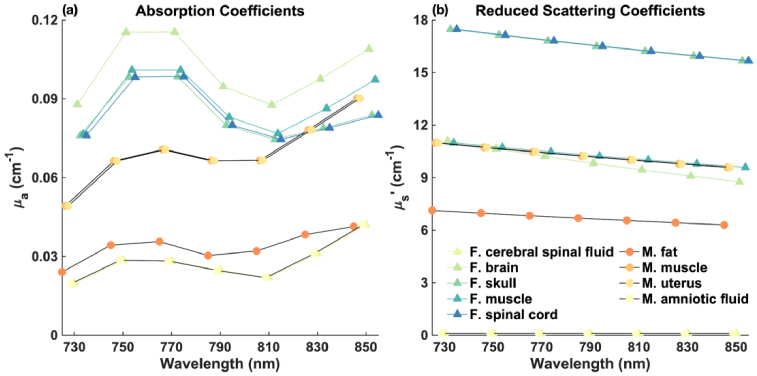
Optical properties of the segmented tissue types across wavelengths. (a) $\mu_{a}$ . (b) $\mu_{s}^{\prime }$ .

Note that for maternal amniotic fluid and fetal cerebral spinal fluid, we consider their $\mu_{a}$ to be equivalent to water, with $\mu_{s}^{\prime }$ set to be 0.1 cm^−1^ for all wavelengths and a refractive index (*n*) of 1.33 [13]. In addition, we assume the optical properties of maternal muscle and uterus are identical. The optical properties of fetal skull and spinal cord are also being treated as equivalent. In this study, we conducted simulations across eight wavelengths: 730, 750, 770, 790, 810, 830, and 850 nm. The $\mu_{a}$ and $\mu_{s}^{\prime }$ for all tissue types across wavelengths are shown in Fig. 3(a-b). For visual clarity, we slightly offset the data points on the x-axis.

#### 2.2.2 Source-detector setup

To comprehensively evaluate the measurement sensitivities across various SD distances and orientations, we placed a 6 × 6 SD grid covering an approximate area of 100 × 70 mm on the surface of the maternal abdomen above the fetal head. Of the total measurement channels formed by 18 sources and 18 detectors, we selected 313 channels with distances ranging from 20 to 120 mm. These channels represent diverse orientations and sensitivities to different tissue depths and regions, and the data collected from these channels will be used for subsequent analyses. The placement of the SD grid is illustrated in Fig. 1(c) for the fetal OA position and in Fig. 2(c) for the OP position.

### 2.3 Data analysis

In this section, we will outline several data analysis methods employed in this study, which include measurement depth sensitivity analysis (2.3.1), imaging depth sensitivity analysis (2.3.2), fetal pulse analysis (2.3.3), dynamic range analysis (2.3.4), and simulated noise model (2.3.5). These methods were applied to 3D models of both OA and OP positions for a comprehensive evaluation.

#### 2.3.1 Measurement depth sensitivity: Jacobian

For each wavelength used in our simulation, we used the “jacobian_FD” function in NIRFASTer to compute the Jacobian ( J ), which a sensitivity matrix. In the context of our continuous-wave domain simulations, Jacobian quantifies how small perturbations in $\mu_{a}$ at each node position (indexed by j ) within the mesh influence the light fluence ( Φ [s^−1^]) measured at each channel (SD pair, indexed by i ) [18]:

$$J_{i,j}=\frac{\partial \Phi_{i}}{\partial \mu_{a,j}}.$$

This analysis enables us to assess the measurement sensitivity of our system to specific tissue types, such as the fetal brain, across different wavelengths and SD distances. To analyze how the Jacobian varies with SD distance, we grouped the 313 measurement channels into intervals ranging from 20 to 120 mm, with increments of 20 mm. Then, a total normalized sensitivity for each node was calculated by summing and normalizing the Jacobian across channels within each SD group:

$$J_{j}^{\text{total}}=\frac{\sum_{i=1}^{\text{NM}}J_{i,j}}{\text{max}\left(\left|\sum_{i=1}^{\text{NM}}J_{i,j}\right|\right)},$$

where *i* and *j* represent SD pair index and node index, respectively, NM is the number of measurement channels within an SD group. This quantity represents the total of all sensitivity matrices calculated for channels within an SD group (numerator), expressed as a percentage of the maximum sensitivity (denominator).

To find the most sensitive channels for fetal brain measurements, we first calculated the total normalized Jacobian for nodes within the fetal brain, and then identified the top ten channels with the highest fetal brain sensitivities for each wavelength and SD group. This selection allows for a focused analysis on the channels that are most crucial for assessing fetal brain signal.

#### 2.3.2 Imaging depth sensitivity: Flat field analysis

While measurement depth sensitivity explores how changes in $\mu_{a}$ affect boundary measurements, imaging depth sensitivity investigates the ability to reconstruct these changes within the tissue based on measured optical signals, particularly those induced by fetal heart pulsations. To assess this capability, we conducted a flat field analysis with the following steps [26–28]:

1. Create a test image by uniformly increasing the $\mu_{a}$ at all nodes within the mesh by 1%. This baseline change allows us to simulate a controlled and uniform perturbation in $\mu_{a}$ across the tissue.
2. Add noise (will be discussed in section 2.3.5) to Φ simulated at baseline and perturbed $\mu_{a}$ , and calculate the ∂Φ due $\mu_{a}$ perturbation: $\partial \Phi =\Phi (\mu_{a,\text{baseline}})-\Phi (\mu_{a,\text{perturbed}})$ .
3. Perform image reconstruction using Moore-Penrose inverse with spatial variant regularization [29]. This step inverts the perturbed optical signals back to potential changes in $\mu_{a}$ :
$$L^{-1}{\tilde{J}}^{T}{\left(\tilde{J}{\tilde{J}}^{T}+I\right)}^{-1}\partial \tilde{\Phi }=\partial \mu_{a},$$
  where *I* is the identity matrix, *L* is the spatial variant regularization defined by:
$$L={\left[\text{diag}(J^{T}J+\alpha )\right]}^{-1/2},\;\text{with}\;\alpha =10^{-2}\cdot \text{max}(\text{diag}(J^{T}J)),$$
  and the adjusted matrices are denoted by:
$$\tilde{J}=\hat{J}M^{-1},\hat{J}=JL^{-1},\text{and}\;\partial \tilde{\Phi }=\partial \tilde{\Phi }M^{-1},$$
  where $M={\left[\text{diag}(\hat{J}{\hat{J}}^{T}+\beta )\right]}^{-1/2}$ , with $\beta =10^{-2}\cdot \text{max}(\text{diag}(\hat{J}{\hat{J}}^{T}))$ .

The reconstructed images highlight the system’s ability to detect and accurately reconstruct small changes in $\mu_{a}$ throughout the tissue, providing important information for optimizing SD configurations. Similar to the approach with the total normalized Jacobian, we summed and normalized the imaging depth sensitivities from all individual channels within each SD group (20 to 120 mm in 20 mm increments). For additional details on the derivation and application of these equations, we refer readers to the foundational works in Refs. [26–28] and [29].

#### 2.3.3 Large detector implementation and fetal signal extraction

To detect small changes in the measured optical density driven by changes in the $\mu_{a}$ due to fetal heart pulsations, particularly at long SD distances, the use of a large detector is crucial. In the simulations, we modeled the response of large detectors by defining a 10 × 10 mm square around each detector location to collect all simulated fluence rates ( ϕ [s^−1^mm^−2^]). This choice of this detector size is supported by recent literature. For instance, in the study by Fong et al. [30] on hardware design for transabdominal fetal pulse oximetry, a 10 × 10 mm photodetector (UDT-555D, OSI Systems, Inc.) was used for their longest SD distance (10 cm). Additionally, Wang et al. [25] performed NIRS measurements for blood oxygenation in the human placenta using a 5 mm core diameter detector (LLG5-8 H, Thorlabs) to ensure sensitivity at a 10 cm distance. Their promising results indicated the effectiveness of larger detectors for this application. Therefore, we adopted a 10 × 10 mm detector in our simulations to explore its potential benefits and provide guidance for future hardware development.

**Fig. 4.**
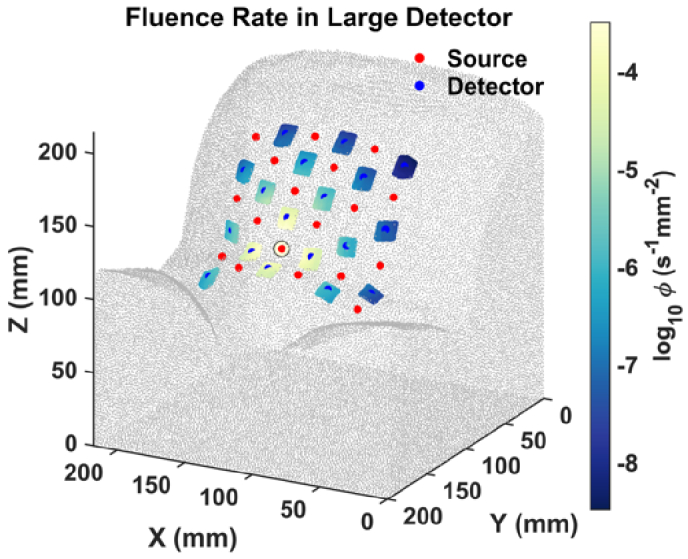
Large detector placement on the maternal abdomen with the fluence rates color-coded to show variations from a single light source, indicated by the yellow circle.

To implement this large detector in the simulation, first, we identified all surface nodes of the tetrahedral mesh within the SD grid. These nodes were then interpolated onto a finer, uniformly spaced mesh, with voxel sizes Δx and Δy both < 0.5 mm in the x and y directions, respectively. At each detector location, we found all nodes within the 10 × 10 mm square, and the fluence ( Φ [s^−1^]) at each large detector was computed based on the number of nodes, *m*, within this area using the formula: $\Phi =\Delta x\cdot \Delta y\cdot \Sigma_{i=1}^{m}\phi_{i}$ . Figure 4 shows an example of large detector placement, with the fluence rates within the detector area color-coded to show variations from a single simulated light source (indicated by a yellow circle).

As described in section 2.2.1, focusing on fetal pulse generation, we conducted two simulations for each wavelength-simulating systolic and diastolic fetal $\mu_{a}$ while keeping the optical properties of the maternal tissues constant. The change in optical density due to the fetal pulse is defined by $\Delta OD=\log(\Phi_{\text{diastole}}/\Phi_{\text{systole}})$ , where $\Phi_{\text{diastole}}$ and $\Phi_{\text{systole}}$ represent the fluence measured from the large detector at diastolic and systolic phases, respectively. We extracted the fetal ΔOD for all wavelengths and SD distance for subsequent analysis.

#### 2.3.4 Dynamic range analysis

Since the fetal brain is deep inside the maternal abdomen, the optical signal resulting from the fetal pulse is exceedingly small. To accurately measure such a subtle signal, a detector with sufficient dynamic range is essential. We calculated the dynamic range using the simulated fluence ( Φ ) measured at the large detector according to the following formula:

$$\text{Dynamic}\;\text{Range}=\frac{\Phi_{\text{diastole}}}{\Phi_{\text{diastole}}-\Phi_{\text{systole}}}$$

This analysis was conducted for all wavelengths and SD distances used in the simulation, allowing us to find adequate dynamic range under each configuration.

#### 2.3.5 Simulated noise model

Incorporating noise into the simulated data is essential for conducting a more realistic sensitivity analysis. Inspired by studies in diffuse optical tomography [26,31], we modeled random white noise that increases with the SD distance. The standard deviation for the Gaussian distribution was generated using the equation $k(r)=0.0024\cdot e^{0.0236\cdot r}$ , where *r* is the SD distance in millimeters. We estimated these noise parameters to approximate for the system used in our study, recognizing that this is a preliminary approach and subject to refinement as more clinical data becomes available. Using this equation, the noise fractions k(r) at SD distances of 20 mm, 60 mm, and 120 mm are approximately 0.4%, 1%, and 4%, respectively, which are within plausible ranges. To add noise to the clean fluence signals, we used the following steps:

1. Generate noisy fluence signals for systolic and diastolic states separately using the equation: $\Phi_{\text{noisy}}=\Phi_{\text{clean}}\cdot (1+N(0,\;k{\left(r\right)}^{2}))$ , where $N(0,\;k{\left(r\right)}^{2})$ represents gaussian noise with mean of zero and standard deviation k(r) .
2. Simulate random noise 480 times (consider a fetal heart rate of 2 Hz, equivalent to 4 minutes of measurement) and average the fluence signal at systolic and diastolic states: $\text{avg}(\Phi_{\text{systole},\;\text{noisy}})$ , $\text{avg}(\Phi_{\text{diastole},\;\text{noisy}})$ .
3. Calculate the noisy ΔOD and dynamic range using the averaged values: $\Delta OD_{\text{noisy}}=\text{log}(\text{avg}(\Phi_{\text{diastole},\;\text{noisy}})/\text{avg}(\Phi_{\text{systole},\;\text{noisy}}))$ , and Dynamic Range $=\text{avg}(\Phi_{\text{diastole},\;\text{noisy}})/(\text{avg}(\Phi_{\text{diastole},\;\text{noisy}})-\text{avg}(\Phi_{\text{systole},\;\text{noisy}}))$ .

Furthermore, the flat field analysis presented in the results section was also performed using these noisy data. This approach allows us to assess the robustness and reliability of our measurements under more realistic conditions.

## 3. Results

### 3.1 Spatial distribution of fluence rate, measurement sensitivity, and imaging sensitivity

**Fig. 5.**
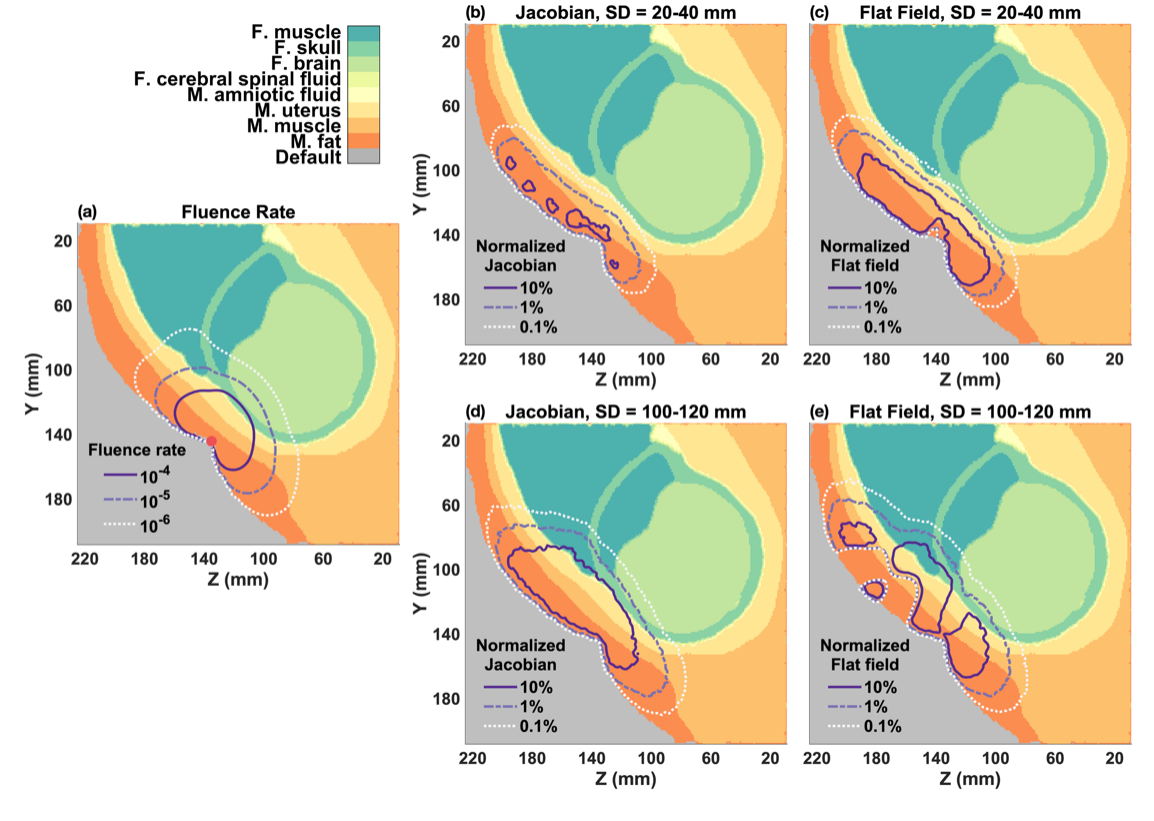
Spatial distribution of fluence rate, Jacobian, and flat field for fetal OA model. (a) Fluence rate distribution from a single light source (indicated by red circle) on the maternal abdomen. (b-c) Total normalized Jacobian and flat field for 20-40 mm SD group. (d-e) Total normalized Jacobian and flat field for 100-120 mm SD group.

In the OA fetal position, the distance between the surface of the maternal abdomen and the fetal brain surface ranges approximately from 25 to 30 mm. Figure 5 illustrates the spatial distribution of fluence rate, measurement sensitivity (Jacobian), and imaging sensitivity (flat field) on a sagittal slice, using a simulation at 730 nm.

**Fluence rate distribution**: As shown in Fig. 5(a), fluence rate decreases rapidly from the light source ( ϕ = 1 s^−1^mm^−2^) on the maternal abdomen, diminishing to ∼10^−4^ to 10^−5^ s^−1^mm^−2^ by the time it reaches the fetal brain surface. This demonstrates the significant attenuation of light as it penetrates deeper tissues.

**Measurement sensitivity analysis**: Fig. 5(b) and (d) demonstrate the normalized Jacobian for SD distances of 20-40 mm and 100-120 mm, respectively. At SD distances of 100-120 mm, the normalized Jacobian at the fetal brain surface slightly exceeds 1%, diminishing to 0.1% just a few millimeters towards the brain center. Conversely, channels with SD distances of 20-40 mm show negligible sensitivity to the fetal brain.

**Imaging sensitivity analysis**: The flat field analysis on noisy ΔOD , shown in Fig. 5(c) and (e), indicates that at the fetal brain surface, normalized flat field sensitivity is between 1-10% for the 100-120 mm SD group, but only around 0.1% for the 20-40 mm group. These sensitivities are small, meaning it’s challenging to reconstruct the change in $\mu_{a}$ from fetal pulse due to fetal depth and noise.

A general rule-of-thumb for effective measurement and reconstruction of changes in $\mu_{a}$ is that the normalized Jacobian should exceed 1%, and the normalized flat field should be around 1-10% [26,32]. These thresholds suggest that, despite low overall measurement sensitivity, it is still feasible to reconstruct changes in $\mu_{a}$ . However, to accurately measure and reconstruct fetal brain signals, particularly those due to heart pulsation, an SD distance of at least 100-120 mm is necessary.

**Fig. 6.**
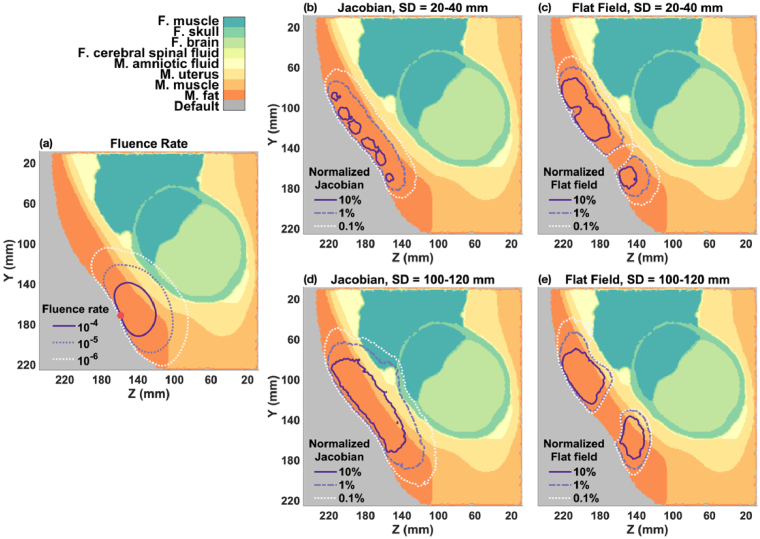
Spatial distribution of fluence rate, Jacobian, and flat field for fetal OP model. (a) Fluence rate distribution from a single light source (indicated by red circle) on the maternal abdomen. (b-c) Total normalized Jacobian and flat field for 20-40 mm SD group. (d-e) Total normalized Jacobian and flat field for 100-120 mm SD group.

**Comparison with OP Position**: For the fetus is in the OP position, the distance from the maternal abdomen to the fetal brain increases to slightly more than 40 mm, approximately 10 mm greater than in the OA case. As shown in Fig. 6(a), the fluence rate at the fetal brain surface (∼10^−6^ s^−1^mm^−2^) decreases further from the light source ( ϕ = 1 s^−1^mm^−2^). Even at longer SD distances (100-120 mm), the normalized Jacobian and flat field sensitivities, shown in Fig. 6(d-e), barely reach 0.1% at the fetal brain surface, indicating that the current setup is unlikely to be sensitive enough to monitor the fetal brain or to reconstruct fetal $\mu_{a}$ changes when the fetal brain is deep (> 40 mm) inside the maternal abdomen.

### 3.2 Highest measurement sensitivity to the fetal brain and the resulting fetal pulse and dynamic range across wavelengths and SD distances

**Fig. 7.**
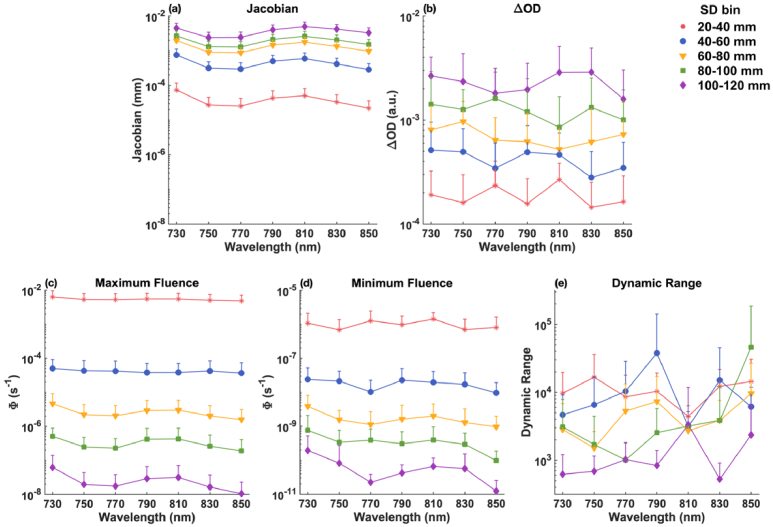
Mean and standard deviation of the best 10 channels within each SD group for the fetal OA position, displayed on a logarithmic scale. (a) Measurement sensitivity (Jacobian). (b) Fetal pulse ( ΔOD ). (c) Maximum fluence ( $\Phi_{\text{diastole}}$ ) measured by the detector. (d) Minimum fluence ( $\Phi_{\text{diastole}}-\Phi_{\text{systole}}$ ) measured by the detector. (e) Required dynamic range (ratio of maximum and minimum fluence) for the detector.

**Fig. 8.**
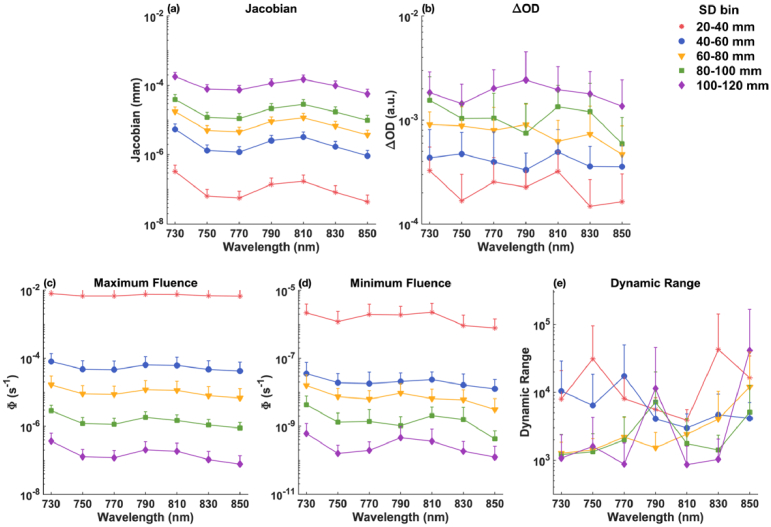
Mean and standard deviation of the best 10 channels within each SD group for the fetal OP position, displayed on a logarithmic scale. (a) Measurement sensitivity (Jacobian). (b) Fetal pulse ( ΔOD ). (c) Maximum fluence ( $\Phi_{\text{diastole}}$ ) measured by the detector. (d) Minimum fluence ( $\Phi_{\text{diastole}}-\Phi_{\text{systole}}$ ) measured by the detector. (e) Required dynamic range (ratio of maximum and minimum fluence) for the detector.

As described in section 2.3.1, for all simulated wavelengths (730, 750, 770, 790, 810, 830, and 850 nm), we calculated the total normalized Jacobian for nodes within the fetal brain, and then identified the top ten channels that have the highest sensitivity to the fetal brain within each SD group (20-120 mm with 20 mm increments). We calculated the mean and standard deviations of measurement sensitivity (Jacobian), and fetal pulse ( ΔOD) and dynamic range that are affected by noise for these channels, depicted in Fig. 7 and Fig. 8 for fetal OA and OP positions, respectively.

**For fetus in OA position**: As illustrated in Fig. 7(a), channels in the 100-120 mm range (represented by purple diamonds) exhibit the highest measurement sensitivity to the fetal brain. Notably, wavelengths at 730 nm and 810 nm show superior sensitivities compared to other wavelengths. This is likely because, as shown in Fig. 3, there is relatively lower absorption at 730 nm and 810 nm. Reduced absorption at these wavelengths allows greater penetration depth of the light, resulting in increased detected fetal signal and improved sensitivity. Figure 7(c-d) show that for the 100-120 mm SD group, the maximum and minimum fluences are about 10^−7.5^ s^−1^ and 10^−11^ s^−1^, respectively, yielding a dynamic range between 10^3^ and 10^4^ across all wavelengths.

**For fetus in OP position**: As shown in Fig. 8(a) and discussed in Section 3.1, channels within the 100-120 mm range are unlikely to be sensitive fetal brain signals, as well as in reconstructing $\mu_{a}$ changes due to fetal pulsation. Figure 8(a) also indicates that the highest measurement sensitivity in the OP position is about one to two orders of magnitude lower than in the OA position across all wavelengths and SD groups. This suggests a significantly reduced fetal pulse measurement from the detector in the OP case, as shown in Fig. 8(b). Although maximum fluence, minimum fluence, and dynamic range are presented in Fig. 8(c-e), it is likely that they primarily reflect sensitivity to other tissue regions or depths rather than the fetal brain.

### 3.3 Optimal channel orientation for the best fetal brain sensitivity

In this section, we visualize the optimal orientations and placements of the channels that demonstrate the highest sensitivity to the fetal brain on 3D maps, aiming to guide real-world clinical setups. Given that the sensitivity across the simulated wavelength range (730-850 nm) is similar (Fig. 7(a)), we selected the 730 nm wavelength for illustrative purposes.

**Fig. 9.**
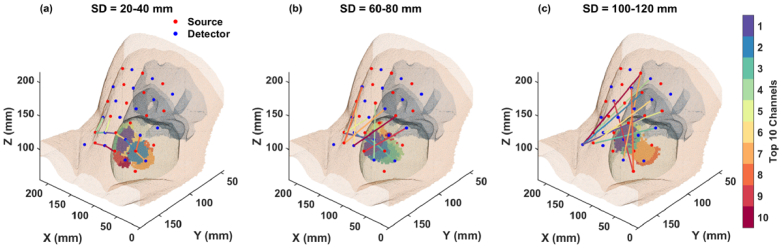
Optimal channel orientations for the fetal OA position. Sensitivities are color-coded from purple (higher sensitivity) to red (lower sensitivity). The shaded areas represent the optimal regions on the brain surface where each channel, indicated by the corresponding color, is sensitive to. (a) Channels in 20-40 mm. (b) Channels in 60-80 mm. (c) Channels in 100-120 mm.

**Fig. 10.**
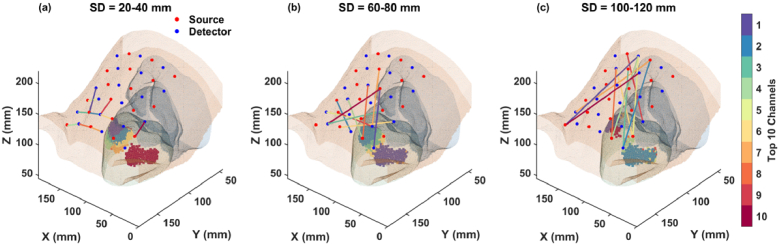
Optimal channel orientations for the fetal OP position. Sensitivities are color-coded from purple (higher sensitivity) to red (lower sensitivity). The shaded areas represent optimal regions on the brain surface where each channel, indicated by the corresponding color, is sensitive to. (a) Channels in 20-40 mm. (b) Channels in 60-80 mm. (c) Channels in 100-120 mm.

Figure 9 and Fig. 10 present the 3D mappings of these channels on the maternal abdomen for fetal positions OA and OP, respectively. From purple (higher sensitivity) to red (lower sensitivity), the SD pairs are color-coded based on their sensitivity levels to the fetal brain, and shaded areas on the fetal brain surface indicate the optimal regions where these channels are sensitive to.

From Fig. 9 and Fig. 10, we can see that the orientation of channels with the highest fetal brain sensitivity varies across different SD groups. Notably, channels that penetrate the fetal brain, especially those crossing the midline of the x-axis and angled along the y-axis, tend to exhibit superior sensitivity. This pattern is observable in the maps for longer SD distances (Fig. 9(b-c) and Fig. 10(b-c)). Additionally, differences in tissue structure and fetal positioning lead to distinct optimal channel configurations between the OA and OP examples, as illustrated by the variations between Fig. 9 and Fig. 10.

This visualization not only highlights the impact of channel orientation and placement on measurement sensitivity but also emphasizes the importance of customizing the transducer array configuration based on specific fetal positioning and anatomical characteristics.

## 4. Discussion

Our simulation study, utilizing realistic geometries derived from MRI scans of pregnant women, offers insights into the optimal configuration for transabdominal fetal pulse oximetry. Our results indicate that a fetal depth of about 30 mm requires a minimum SD distance of 100 mm to effectively measure and reconstruct changes in $\mu_{a}$ due to fetal heart pulsation. Additionally, our results indicate that among the simulated wavelengths from 730 to 850 nm, 730 nm and 810 nm exhibit higher sensitivities to the fetal brain. For optimal separation from the isosbestic point of oxy- and deoxy-hemoglobin, wavelengths of 730 nm and 830 nm are recommended. In addition, the 3D mappings of optimal channel orientations on the maternal abdomen (Fig. 9 and Fig. 10) suggest that customizing the transducer array configuration based on individual fetal positioning and anatomical characteristics can maximize sensitivity to the fetal brain. Alternatively, employing a high-density SD setup that covers a large area atop the fetal head could also enhance sensitivity by incorporating diverse channel orientations. In this setup, the shorter SD distances would primarily be sensitive to maternal tissue, while the longer SD distances would be proportionally more sensitive to the fetus. The fetal signal could be better extracted by subtracting the signal measured at shorter SD distances from that at longer distances [33,34].

Previous studies have primarily utilized simplified geometries like slab or spherical models. For instance, Böttrich et al. [11] showed that optimal SD distances vary significantly with model geometry due to differences in photon propagation and signal composition. In their study, the slab layer model showed that a SD distance of about 140 mm optimizes the mixed fetal-maternal component’s contribution. Conversely, the spherical layer model (radius = 15 cm) identified an optimal distance of 85 mm, where a ‘hot spot’ demonstrates the significant impact of maternal abdomen curvature on photon propagation and signal composition. These findings underscore the importance of employing realistic geometries, as they provide more accurate and clinically relevant insights.

In addition to geometric considerations, the simulation study by Fong et al. [13] explored optimizing SD distance and wavelengths using a multi-layered slab model and Monte Carlo simulations. They emphasized the importance of considering the noise limits of the electrical component as increasing the SD distance improves sensitivity to the fetal layer but also reduces the overall signal strength, potentially falling below the minimum detectable power of the detector. On the other hand, our study focused on sensitivity analysis using the Jacobian matrix and incorporated a simulated noise added to the measured intensity, with magnitude increasing with SD distance. However, future simulations could benefit from integrating realistic noise models derived from clinical experiment with specific detector specifications. This approach could help refine SD distance and hardware selection for better clinical implementations.

Our simulations, using continuous-wave NIR light, indicate limitations in depth sensitivity, echoing the need for advanced hardware systems. Sassaroli et al. [35], Blaney et al. [36], and Fan et al. [37] demonstrate how the dual-slope method and frequency-domain near-infrared spectroscopy (FD-NIRS) can improve measurement depth sensitivity by utilizing the phase data. Likewise, Liu et al. [38] introduced interferometric NIRS (iNIRS), showing significant advantages in depth sensitivity and fetal signal extraction compared to using continuous-wave NIR light. These findings suggest the potential of FD-NIRS and iNIRS to surpass the limitations of conventional light sources used in pulse oximetry, enhancing the efficacy of non-invasive fetal monitoring.

For transabdominal fetal pulse oximetry, it is critical to separate the fetal dynamics from the maternal dynamics, which include heart rate, respiration, uterine contractions, etc. Advanced signal processing techniques, as demonstrated in various studies, have shown success in differentiating these dynamics. For instance, Zahedi et al. [39] used optical signals measured with photoplethysmography (PPG) from the maternal abdomen and employed adaptive filtering and a recursive least-squares algorithm to isolate fetal heart rate with high accuracy. Behar et al. [40] reviewed methods for fetal heart rate extraction using fetal electrocardiogram (ECG), summarizing practical filtering techniques. Beyond conventional signal processing methods, deep learning approaches have also been effective—Fotiadou et al. [41] demonstrated that accurate fetal heart rate can be extracted from fetal ECG, with preserved beat-to-beat variations. Combining fetal ECG and fetal PPG, Kasap et al. [42] showed significant improvement in extracting fetal signals compared to using fetal ECG or fetal PPG alone. These techniques leverage the distinct frequency characteristics and temporal patterns of fetal and maternal dynamics to achieve separation. Incorporating such advanced signal processing methods can significantly enhance the accuracy of transabdominal pulse oximetry measurements by accurately extracting fetal signals.

In addition to transabdominal fetal pulse oximetry, other modalities have been explored to enhance maternal and fetal outcomes. Hutter et al. [43] used MRI to study functional changes in the placenta, which could help optimize the timing of interventions to prevent fetal injury. Wang et al. [25] combined FD-NIRS and ultrasound imaging to measure placental tissue oxygen saturation, similar to our study, using a source-detector distance of 10 cm to probe the placenta at a depth of about 3-4 cm. Recent advances in photoacoustic imaging have also shown promise for direct measurement of fetal brain oxygenation [44]. As these technologies mature, they may provide a powerful approach to improve perinatal care by enabling more comprehensive monitoring of placental function, fetal oxygenation, and brain development.

While our study provides a foundational understanding, it is limited by the small number of fetal positions considered and does not capture the full range of anatomical variability found in clinical practice. Additionally, our segmentation process did not encompass the full complexity of anatomical structures. Future studies should incorporate a broader spectrum of fetal positions and maternal anatomies, along with varying tissue optical properties and more realistic fetal pulsation models, to enhance the generalizability and clinical relevance of our simulation results. Accurately determining the influence of other tissue regions on the measurement sensitivity is also important, but it’s complicated due to the non-uniform nature of each tissue layer and the varying sensitivity of different source-detector channels to different tissue depths and volumes. Future research could use ultrasound-guided source-detector placement to enhance the sensitivity to the fetus by ensuring optimal positioning of the detectors. Furthermore, future research should quantify the improvement and necessity in signal acquisition by using a larger detector, as different detector sizes impact signal quality and device compactness. This will help determine the optimal detector size, balancing signal acquisition efficiency with practical design constraints.

## 5. Conclusion

In conclusion, our study utilized realistic 3D models derived from MRI scans of pregnant women to explore optimal SD configurations for transabdominal fetal pulse oximetry. Our findings demonstrate that, despite challenges, it is theoretically feasible to detect fetal cardiac pulses for pulse oximetry at a fetal depth of 30 mm using an SD distance greater than 100 mm and wavelengths between 730 and 850 nm. As hardware and signal processing technologies continue to advance, transabdominal fetal pulse oximetry has the potential to become a standard practice in fetal monitoring. This advancement could significantly improve maternal and fetal outcomes by minimizing unnecessary interventions and enhancing the early detection of fetal distress.

## Data Availability

Data and code will be made available at [45].
